# Policies on return and reintegration of displaced healthcare workers towards rebuilding conflict-affected health systems: a review for *The Lancet*-AUB Commission on Syria

**DOI:** 10.1186/s13031-021-00367-4

**Published:** 2021-05-07

**Authors:** Diana Rayes, Lana Meiqari, Rouham Yamout, Aula Abbara, Iman Nuwayhid, Samer Jabbour, Marian Abouzeid

**Affiliations:** 1 The Lancet-AUB Commission of Syria, Faculty of Health Sciences, American University of Beirut, Beirut, Lebanon; 2 Johns Hopkins Bloomberg School of Public Health, Baltimore, USA; 3 Vrije Universiteit Amsterdam, Amsterdam, Netherlands; 4 Imperial College London, London, UK; 5 Alfred Deakin Institute for Citizenship and Globalisation and Centre for Humanitarian Leadership, Deakin University, Geelong, Australia

**Keywords:** Displacement, Healthcare workforce, Return, Reintegration, Human resources for health, Post-conflict health systems

## Abstract

**Background:**

War and armed conflicts severely disrupt all health system components, including the healthcare workforce. Although data is limited on the scale of health care worker (HCW) displacement in conflict zones, it is widely acknowledged that conflict conditions result in the displacement of a significant portion of qualified HCWs from their country of origin. While voluntary HCW return is integral to health system rebuilding in conflict-affected and post-conflict settings, there has been little exploration of the nature of national or international policies which encourage HCW return and reintegration to their home countries in the post-conflict period.

**Methods:**

We conducted a systematic review to identify policies and policy recommendations intended to facilitate the return of displaced HCWs to their home countries and acknowledge their contribution to rebuilding the post-conflict health system. We searched three bibliographic databases and a range of organisational and national health agency websites to identify peer-reviewed articles and grey literature published in English or Arabic between 1 January 1990 to 24 January 2021, and extracted relevant information. We classified policies and policy recommendations using an adapted version of the UNHCR 4Rs Framework.

**Results:**

We identified nine peer-review articles and four grey literature reports that fit our inclusion criteria, all of which were published in English. These covered issues of repatriation (*n* = 3), reintegration (*n* = 2), health system rehabilitation and reconstruction (n = 2); six documents covered several of these themes. Information was available for nine conflict contexts: Afghanistan, Iraq, Kosovo, Lebanon, Namibia, Northern Uganda, South Sudan, Timor Leste, and Zimbabwe. Findings demonstrate that health system rebuilding and rehabilitation serve as precursors and reinforcers of the successful return, repatriation, and reintegration of displaced HCWs.

**Conclusions:**

Despite the significant numbers of HCWs displaced by conflict, this study identified few specific policies and limited information explicitly focused on the repatriation and reintegration of such workers to their home country in the post-conflict period. Additional research is needed to understand the particular barriers faced by conflict-displaced HCWs in returning to their home country. Conflict-affected and post-conflict states should develop policies and initiatives that address factors within and beyond the health sector to encourage displaced HCW return and provide sustainable reintegration solutions for those who return to post-conflict health systems.

## Background

By the end of 2019, an estimated 80 million people were forcibly displaced by conflict, persecution, and threats to livelihood, of whom 30.2 million (37%) are considered refugees or asylum seekers [1]. Although there is little data on the number of healthcare workers (HCW) who are among those forcibly displaced or who have emigrated as a result of conflict, it is widely acknowledged that the loss is substantial, with profound impacts for the performance and recovery of health systems weakened by conflict [2–4]. For example, it is estimated that almost two-thirds of all HCWs and 70% of doctors have been forcibly displaced from Syria since the conflict onset [5, 6]. Similarly, in Iraq, a third of the country’s HCWs have fled since the 1990s [2, 7]. Many factors may drive HCW displacement, *push factors* include threats or targeting by armed actors [2], criminalisation for provision of medical care [2], and pressures to deliver healthcare in facilities that are under-staffed, under-resourced, or are subject to deliberate and targeted attacks [3, 5]. *Pull factors* to new countries include personal and professional connections abroad, career opportunities and financial rewards, and personal security and safety considerations [8].

The World Health Organization (WHO) estimates a shortfall of 18 million HCWs by 2030, mainly in low- and lower-middle income countries [9, 10]. In conflict-affected and post-conflict settings, HCW shortages will remain more pronounced with detrimental short- and long-term consequences on the provision of and access to health services and as a result, on population health status [8]. Prolonged HCW shortages and associated protracted health system disruptions can in turn fuel conflict and contribute to the continued displacement of civilian populations in search of areas with adequate healthcare coverage [10, 11]. Ultimately, this can compromise social, economic, and political recovery and development efforts, particularly in low- and middle-income countries [12].

In 2010, the WHO adopted a Code of Practice for Health Worker Migration, issuing guidelines on ethical practices in the international recruitment of health personnel, including the protection of human resources for health in lower income countries with fragile health systems [13, 14] and set standards to prevent the “active recruitment of health personnel from developing countries facing critical shortages of HCWs.” However, little attention has been paid to practices, including ethical ones, in which displaced HCWs could be incentivized to return to their home countries to contribute to health system rebuilding in the post-conflict phase [8, 15].

Researchers have attempted to quantify the magnitude, nature and impact of healthcare workforce shortages, as well as understand the push and pull factors that contribute to “medical brain drain” and “out-migration” of HCWs [13–17]. In post-conflict settings, initiatives to fill HCW gaps have included scaling-up in-country training and recruitment; managing deployment, incentivisation, and redistribution of non-displaced and *internally*-displaced HCWs; analysing and adjusting policy mechanisms which support task-shifting and/or skill substitution; as well as encouraging retention of HCWs in the first instance [18–27].

To our knowledge, there has not been a synthesis of policies or interventions developed by state and non-state actors, at either national or international levels, regarding the return and reintegration of *externally*-displaced HCWs to their home countries in the post-conflict period and to support their role in health system rebuilding [28–30]. Such information is of considerable contemporary relevance, particularly in considering health workforce rebuilding in increasingly protracted current conflict settings such as Syria and Yemen that have witnessed massive exodus of HCWs and widespread destruction of their national health systems [2, 31]. Lessons learned from past conflict contexts may potentially help inform policy development for health systems currently in the throes of conflict and post-conflict transition.

The aim of this study is to identify policies and policy recommendations developed to facilitate the return of externally-displaced HCWs to their home countries and encourage their contribution to the rebuilding of the post-conflict health system.

## Methods

This review follows the Preferred Reporting Items for Systematic Reviews and Meta-Analyses (PRISMA) guidelines [32].

### Key definitions

We searched for peer-reviewed and grey literature identifying policies as defined by the WHO as “Decisions, plans, and actions that are undertaken to achieve specific healthcare goals within a society.” [33] We defined HCWs were defined according to the WHO’s occupational classification for health professionals and health associate professionals, which largely draws on the International Standard Classification of Occupations [34, 35]. Community HCWs (CHWs) were also included in this definition.

### Search strategy

We searched for both English and non-English peer-reviewed and grey literature published between 1 January 1990 and 24 January 2021 across three electronic databases (PubMed, Embase, Web of Science). Search terms included a combination of keywords and index terms to capture the main elements of the review, including: (1) health care/healthcare workers (e.g., “health personnel,” “health workforce,” “doctor”); (2) conflict (e.g., “armed conflict,” “warfare,” “post-conflict”); (3) return, integration, and reconstruction. The full search strategy is available in the Appendix. To identify relevant grey literature, we searched websites of United Nations (UN) agencies (UN High Commissioner for Refugees, WHO, UN Children’s Fund, International Organization for Migration, UN Population Fund, International Labour Organization); humanitarian information platforms (including ReliefWeb, Active Learning Network for Accountability and Performance, ReBUILD Consortium, Human Resources for Health Global Resource Center, the Information Center about Asylum and Refugees); other humanitarian agencies (including Médecins Sans Frontières, International Committee of the Red Cross, Médecins du Monde, Syrian American Medical Society, Physicians for Human Rights, European Council on Refugees and Exiles, and the Refugee Council); and national health departments for selected countries that have recently experienced conflict in order to identify any English- or Arabic-language national policy documents related to HCW return. For both peer-reviewed and grey literature searches, we used a snow-pooling strategy which involved the screening of the reference lists of included records to identify additional references.

### Eligibility criteria

Peer-reviewed articles were included if full-texts were available in English or Arabic and met the following criteria  (a) mention of a post-conflict setting(s), defined as a conflict that took place within the last three decades (since 1990), (b) focused on externally-displaced HCWs, defined as HCWs engaged in clinical practice before conflict onset who were externally-displaced or had migrated from their country of origin due to conflict, and (c) described policies implemented in order to encourage return of externally-displaced HCWs and their contribution to the rebuilding of the health system of their home country in the transitional and post-conflict stages. All study designs and publication types were considered.

We excluded records  that a) addressed internally-displaced HCWs or those in the diaspora before conflict onset, b) did not focus on HCWs (e.g. lay volunteers or researchers), c) did not mention a post-conflict setting, and d) did not implicitly or explicitly describe policies issued in post-conflict-contexts regarding the return of HCWs to their home countries. 

### Selection process

We selected records through a multi-step process. One reviewer (LM) screened titles and abstracts using Rayyan QCRI [36]. Two reviewers (DR and RY) independently reviewed full texts of retained articles using Covidence [37], with disagreements resolved by discussion and input from other authors when needed. One reviewer (DR) identified and screened relevant grey literature against the inclusion criteria.

As this review focused on identifying and summarizing policies regarding HCW return through various publication types and did not seek to evaluate the effects and outcomes of such policies, no quality assessment was performed.

### Data extraction and analysis

For each record, we extracted relevant information including bibliometrics, study characteristics (including design, location, objective, time period, conflict under study), relevant concepts (including type of HCWs, conflict phase, and type of return), detailed description of related policy, challenges reported in implementation, and lessons learned. Where available, we summarized the outcomes of the policy or initiative, as described by study or report authors.

To categorise these findings, we used the United Nations High Commissioner for Refugees (UNHCR)‘s 4Rs Framework for Durable Solutions for Refugees and persons of Concern, which considers four elements: repatriation, reintegration, rehabilitation and reconstruction [38], and adapted the framework definitions to externally-displaced HCWs (Table 1). Themes were synthesised and summarised narratively. For the purpose of our analysis, rehabilitation and reconstruction of the health system were considered together.

**Table 1:** Adaptation of the UNHCR 4Rs Framework [38] for externally-displaced HCW populations

The 4Rs Framework and Definitions	
Repatriation	Free, voluntary return of HCWs to their country of origin in safety and dignity.
Reintegration	Ability of returned HCWs to secure the necessary political, economic, legal and social conditions to maintain their lives, livelihoods and dignity.
Rehabilitation	Restoration of social and economic health infrastructure (e.g. clinics, hospitals, schools, colleges, and universities) destroyed during conflict to enable HCWs to pursue sustainable livelihoods upon return to their country of origin.
Reconstruction	Re-establishment of political order, institutions and productive capacity to create a base for sustainable development for returned HCWs.

## Results

### Records selection and characteristics

**Fig. 1:**
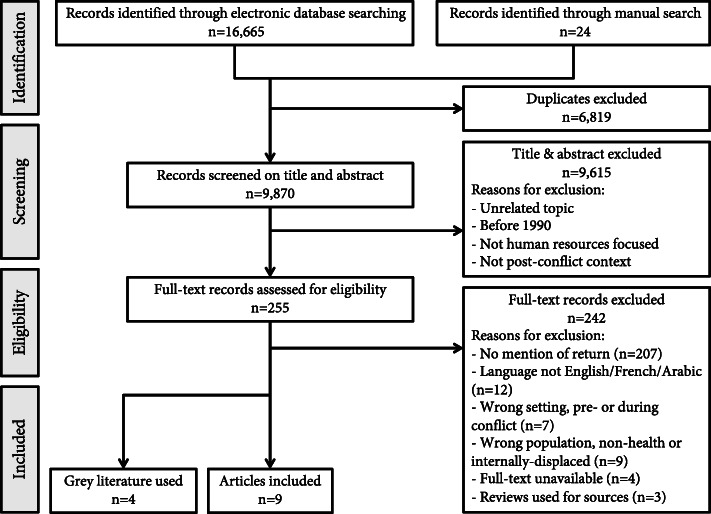
Summary of search results and record handling using PRISMA guidelines

Of 9870 unique records identified in our search, a total of nine peer-reviewed papers and four grey literature sources met the inclusion criteria (Fig. 1).

Tables 2 and 3 present an overview of the peer-reviewed articles and grey literature reports, respectively. These covered numerous conflict and post-conflict contexts, including Timor-Leste (*n* = 1) [39], Afghanistan (*n* = 1) [40], Zimbabwe (*n* = 1) [41], South Sudan (*n* = 3) [42, 44, 48], Lebanon (*n* = 1) [43], Namibia (*n* = 1) [45], Kosovo (*n* = 1) [46], Iraq (*n* = 1) [47] and northern Uganda (*n* = 1) [15]. Two records examined multiple contexts [49, 51].

**Table 2:** Summary of key characteristics of peer-reviewed articles, by thematic focus

First author, publication year	Article type/study design	Description	Study setting (study period)	Type of HCW
Multiple themes				
Bertone, 2018 [39]	Qualitative	Reports policies and policy-making processes at central level related to healthcare workforce recruitment and presents a political economy analysis about how and why both official and informal practices developed, as well as the drivers, challenges and blockages at different stages.	Timor-Leste (1998–2018)	Multiple (with a focus on nurses)
Bristol, 2005 [40]	Commentary	Identifies challenges faced by expatriate doctors returning to Afghanistan to provide essential healthcare	Afghanistan (2001–2005)	Multiple (i.e. Expatriate doctors based in the U.S., midwives, community HCWs)
Chikanda, 2006 [41]	Qualitative	Assesses the magnitude of the migration of skilled health personnel, analysed the effects on health care delivery, and recommended ways of retaining skilled health personnel.	Zimbabwe (2002)	Multiple (Individual HCWs, migrant health staff, and returnee health staff)
Crutcher, 2008 [42]	Case Study	Describes the Sudanese Physician Reintegration Programme.	South Sudan (2008)	Physicians (Sudanese physicians)
Repatriation of externally displaced HCWs				
Akl, 2008 [43]	Observational	Examines whether the repatriation of Lebanese physicians educated abroad has contributed to the international emigration of recent Lebanese medical graduates.	Lebanon (1977–2006)	Physicians
Finlay, 2011 [44]	Review	Examines the motivations and factors influencing the planned return to Sudan of the 15 participants in the Sudanese Physician Reintegration Programme, using a multiple life history approach	South Sudan (2006)	Physicians (Qualified Sudanese physicians trained in Cuba)
Reintegration of returned HCWs				
Preston, 1994 [45]	Case Study	Analyses the situation of people seriously affected by the war of independence and the effectiveness of policy initiatives or their own efforts to facilitate their integration into society.	Namibia (1993)	Multiple
Rehabilitation and reconstruction of the health system				
Morikawa, 2003 [46]	Case Study	Describes a training program by both expatriate and returned staff that supported the reintegration of returned staff	Kosovo (1999)	Multiple (GPs and nurses)
Al Hilfi, 2013 [47]	Review	Describes the health status of Iraqis, the function of Iraq’s health system, the rapid changes occurring in the health sector, and the need for improved policies to guide these processes	Iraq (2003–2011)	Multiple

**Table 3:** Summary of key characteristics of grey literature articles, by thematic focus

Title	Description	Source	Publication Type	Setting (location)	Type of HCW	4Rs Theme
The long road home: Opportunities and obstacles to the reintegration of IDPs and refugees returning to Southern Sudan and the Three Areas	Presents the findings from the second phase of an in-depth research project on the reintegration of IDPs and refugees returning to Southern Sudan and the Three Areas and delimits strategies to facilitate successful reintegration, outline the roles different actors (government, returnees, host communities, donor governments and aid agencies) should play and develop models applicable to other parts of South Sudan.	ODI, 2008 [48]	Report	Southern Sudan	Multiple	Reintegration of returned HCWs
Policies to attract and retain HCWs in Northern Uganda during and after conflict: findings of key informant interviews	Outlines the evolution of government and donor policies supporting health workers during and after the conflict in Northern Uganda, and to derive recommendations on how to improve their effectiveness and sustainability.	ReBUILD, 2014 [15]	Report	Northern Uganda	Multiple	Repatriation of externally displaced HCWs
Guide to healthcare workforce development in post-conflict environments	Provides evidence on the issue of healthcare workforce shortages on a global level and uses examples to illustrate strategies in order to overcome this crisis in post-conflict contexts.	WHO, 2005 [49]	Guide	Multiple	Multiple	Multiple
World Health Report: Working Together for Health	Draws attention to the crucial importance of human resources development for re-establishing health systems following prolonged periods of conflict and disruption; provides evidence and tools to de facto health authorities and other actors in the field of human resources to support them in their difficult task of post-conflict reconstruction; encourages de facto health authorities, donors and nongovernmental organizations to share knowledge and experience which can be shared with those in the field.	WHO, 2006 [50]	Report	Multiple	Multiple	Multiple

Included peer-reviewed articles were published between 1994 and 2018, covering study periods from 1970 to present day. Almost half of the articles had a case-study design. Grey literature findings were published between 2005 and 2014 and included a variety of reports and briefings on the topic of HCW displacement.

## Main themes

### Repatriation

Four peer-reviewed articles discussed the repatriation of externally-displaced HCWs for Afghanistan [40], South Sudan [42, 44] and Lebanon [43], with return described as voluntary and independent from official or governmental encouragement.

In a case study from Afghanistan, the Minister of Public Health appealed to expatriate Afghan physicians in the U.S. to return to Afghanistan in order to train local physicians as well as provide essential services to their fellow citizens [39]. .This is supported through the distribution of public surveys by the Ministry of Public Health (MoPH) in Afghanistan in order to map the existing Afghan medical diaspora and gauge their interest in supporting the Afghan health system [52].

In South Sudan, two articles discussed the Sudanese Physician Reintegration Program (SPRP), a voluntary return assistance program hosted by the University of Calgary and Samaritan’s Purse Canada [42, 44]. The objective of SPRP was to facilitate the repatriation of Sudanese-Canadian physicians to South Sudan. The program provided returning Sudanese physicians with short-term support in the form of education, training, living costs, medium to long-term assistance that enabled physicians to return to South Sudan, and expedited the Canadian citizenship process in order to allow participants to travel freely to Sudan and potentially return to Canada, as needed [42]. Important aspects of this program also included the identification of a Sudanese diaspora in Cuba who were motivated to return to their country of origin and willing to participate in refresher training at a Canadian university in order to prepare for their return to a post-conflict context [42, 44]. Out of the original 15 physicians who participated in the SPRP program, a total of 11 were able to work in remote areas across South Sudan and have contributed to the expansion of secondary and tertiary health service access and availability for local communities [42].

In Lebanon, the repatriation of externally-displaced HCWs who had received additional training abroad had an unintended impact on the local healthcare workforce, including younger generations of HCWs who were searching for employment opportunities in a competitive, post-conflict environment [43]. Akl et al. reported that “the repatriation of Lebanese physicians educated abroad has contributed to the international emigration of recent Lebanese medical graduates”, and recommended the development of a comprehensive national healthcare workforce plan to address the return of externally-graduated or expatriated HCWs which included “defining the optimal size of the physician workforce, devising strategies to reach this size, and improving local residency training programs” in order to prevent and mitigate potential consequences on existing HCWs in post-conflict settings [43].

### Reintegration

Citing examples from different contexts, a guide published by WHO on healthcare workforce development in post-conflict settings found that there existed a variety of “ambiguity and political sensitivities to reintegrating HCWs in post-conflict contexts from different political sects/ factions returning to fractured communities.” [49] Similarly, a report published by the ODI  emphasized the importance of collaborating with the MoH to conduct assessments about HCW distribution, as well as provided recommendations regarding how best to coordinate the response for return and reintegration of all staff, including health staff [48]. In a case study from South Sudan, for example, a total of 436 externally-displaced HCWs had been reintegrated into the public health system following the end of conflict in South Sudan [48].

Several factors influencing recruitment and reintegration of returned HCWs to the post-conflict job market were also described, including logistical and financial barriers, legal status and certification, and the relationship between displaced HCWs and HCWs who stayed behind.

#### Logistical and financial barriers to reintegration

 Various examples of barriers that prevent or inhibit the successful reintegration of externally-displaced HCWs were  described, including the lack of sustained financial and logistical support, or  incentives, for returning HCWs. In Zimbabwe, over 80% of key informants interviewed by Chikanda et al. said that higher salaries would motivate skilled HCWs to return back to their country of origin, while nearly 60% of informants mentioned better incentives as major pull factors for externally-displaced HCWs [41]. Bristol et al. identified challenges faced by the MoPH in Afghanistan to encourage the return of Afghan physician expatriates from the United States (U.S.), including the regular and sustained sources of funding to support the provision of temporary housing and adequate salaries for HCWs returning to participate in a year-long placement program [40]. Other types of incentives, such as  the availability of functioning health facilities equipped with medical supplies and medication for HCWs to return to, are referenced under the following theme on rehabilitation and reconstruction of the health system.

#### Legal status and certification

One example from Timor-Leste demonstrated the importance of salvaging records of health personnel in order to track those who have left as well as verify certification for future recruitment [39]. Drawing on lessons learned from a variety of contexts, a WHO report suggested an expansion of HCW registration on a global level (via the International Organization for Migration-IOM), including a database with the Ministry of Health regarding work experience and qualifications of HCWs, including diaspora HCWs to identify skilled potential returnees [51].

#### Relationships with HCWs who stayed

Articles documented feelings of resentment towards returning HCWs felt by colleagues who did not leave during the conflict. For example, in Afghanistan, administrators noted emotional resentment from HCWs who did not leave during the conflict towards “Westernized” professionals who returned, noting that “working through those relationships is tricky.” [39] An example from Namibia claimed that fear of “well-educated returnees,” including health professionals, was “unfounded as formal qualifications are not matched with work experience.” [45] Nonetheless, those who return to Namibia are noted to be likely more educated, and therefore, will have higher chances of finding employment.

### Rehabilitation and reconstruction of the health system

Within the themes of rehabilitation and reconstruction of the health system, several sub-themes were identified:

#### Strengthening of the health system to facilitate return and prevent additional brain drain

The quality of the post-conflict health system plays a significant role in the willingness and capacity of HCWs to return. Limited or lack of health system reconstruction in a post-conflict context can also lead to repeated brain drain, which can hinder the rehabilitation of the health system due to poor financial management, lack of access to information and incentivizing working environments, which may create the push for HCWs to leave their home country once again [47].

Chikanda et al. identified several health system-related factors in Zimbabwe that could contribute to the return of HCWs who are abroad; namely, good working conditions, such as the availability of adequate drugs and equipment, and a well-developed human resources policy [41]. Other articles described both successes and failures of attempting to indirectly increase retention of HCWs through health system strengthening. Al Hilfi et al. provided the example of Iraq, where efforts to motivate migrant Iraqi doctors in the U.S., United Kingdom, and Australia to return have been unsuccessful, suggesting that, “an efficient, well-functioning health system could go a great way to stabilise this professional drain.” [47] Consequently, unsuccessful efforts to recruit Iraqi migrant physicians back resulted in efforts to improve employment options for existing physicians and other types of HCWs, including immediate employment through the Iraqi Ministry of Health post-graduation and filling gaps between rural and urban areas, based on HCW preference [47].

In Northern Uganda, faith-based organizations, including churches, played an influential role in mobilizing expatriated physicians (including both Ugandans and other nationalities) to return and contribute to rebuilding the health system [27]. Key informant interviews conducted by the ReBUILD Consortium noted the challenges of motivating externally-displaced Ugandan doctors to return due to fear of possible re-emergence of conflict [15]. This resulted in the recruitment of expatriate physicians to provide short-term solutions for health system rebuilding, which can indirectly push out or create fewer opportunities for HCWs willing to return in the long run.

#### Training and educational initiatives

The training and up-skilling of returned HCWs can contribute to health system rebuilding and may be contributing factors to return. In interviews with HCWs from Zimbabwe, Chikanda et al. reported that 17% of skilled migrant HCWs would return to their country of origin if prospects for further education existed [41]. One article described an effort led by German and American physicians to train four Albanian physicians and five nurses during the conflict while they were externally-displaced in a refugee camp in Macedonia [46]. The training focused on “practical bedside training and moral support” throughout both conflict and post-conflict periods. These trained HCWs returned to Kosovo following the conflict and reopened a clinic that provided healthcare services in a post-conflict context. The program was utilized by WHO as a useful model to set up a 2-year formal family practice training program to support HCWs and reorganize primary care services across the Kosovo region [46]. In Timor-Leste, the Ministry of Health requested support from Cuba to train over 1000 Timorese doctors, who were then expected to return to serve in the national public health sector upon graduation [39].

#### Role of returnee HCWs

A number of studies demonstrated that returning HCWs can be particularly useful in providing support to areas that are underserved in post-conflict settings. For example, Sudanese physicians who completed their training in Canada and returned to South Sudan were effectively employed to work in rural areas with massive labour shortages upon their return [42, 44]. The essential role of returning HCWs was also echoed in a WHO report which highlighted a ‘repair and prepare’ strategy that involved the redeployment of HCWs who had fled to areas that had a shortage of staffing and could be described as most vulnerable areas [49]. In Kosovo, returned HCWs reopened a clinic to provide direct care to their community. Additionally, returnees can be involved in the training of the local staff [46].

## Discussion

This review demonstrates the paucity of information regarding policy levers to encourage the return of displaced HCWs and health workforce rebuilding in post-conflict settings. Furthermore, our search did not identify any explicit programs or initiatives focused on this issue. The limited literature available points to several barriers to return and identifies a number of potential entry points for future policy-making.

Particularly among HCWs who fled war and conflict, the decision to return is complicated by ongoing political and security conditions of the conflict-affected country. Included articles suggest that the drivers of HCW displacement are more strongly influenced by push factors from conflict-affected settings rather than pull factors from receiving countries, trends which are substantiated by research on economic migration of HCWs [48]. Examples of push factors demonstrated in our findings include ongoing political or military violence, unsafe working conditions, low pay, and limited opportunities for continued education or professional development [40, 41, 47]. Based on our limited findings, identifying and addressing issues which drive displacement in the first instance are key to creating an environment conducive to return. This provides grounds for the close alignment of health, political action and social policies which emphasize the security and sustainable livelihoods of returning HCWs, and indeed all displaced populations.

Some of the reported drivers of repatriation in this review were the personal will of HCWs to return home after a long exile in order to see family and friends [40, 44], including feelings of responsibility to contribute their skills in rebuilding their country’s health system and provide capacity-building for the national healthcare workforce [40, 44], or the opportunity to seek better work conditions in a context of market regulation [43]. For example, in a survey conducted with key informants in Zimbabwe, almost half of the respondents (41.7%) named a stable political climate as a key factor to influence the return of HCWs who are abroad, in addition to redress of macro-economic environment (16.7%) [41]. Among displaced South Sudanese physicians in training in Canada, the factors that impeded their return to Sudan included “waiting for the right circumstances” or the financial means to do so, while facilitating factors for return included the prospect of reuniting with family, returning to Sudan “to complete their mission” in rebuilding South Sudan following the end of the civil war, as well as contributing their skills to a country that was experiencing a shortage in skilled medical professionals [44].

Experiences in host countries also shape decisions regarding return. There are some examples that HCWs who returned to their countries of origin were more likely to have received training or support from their respective host countries; for example, a successful reintegration program for physicians [42] facilitated the return of Sudanese physicians from their training in Canada to South Sudan [42, 44]. While this program was funded by private donors and public NGOs, government-funded programs specifically targeting the health sector can potentially result in greater numbers of returns and thus be more impactful. Such return assistance programs can follow the model of the IOM Assisted Voluntary Return and Reintegration programs for displaced populations more broadly, which provide financial and technical support for individuals who want to return home but lack the means to do so [50]. Such programs may succeed because of “the alignment of returnee commitment and motivation to return with significant and sustained support,” and have been recommended in other post-conflict contexts [44, 53].

A number of our studies indicate that the reconstruction and rehabilitation of the health system serve as precursors as well as reinforcers of the successful return, and reintegration of conflict-displaced HCWs. This was demonstrated in Zimbabwe where HCWs indicated the importance of good working conditions, availability of healthcare resources, and human resource policies would incentivize return [41]. A case study from Iraq also showcased how difficult it was to recruit displaced doctors to support an inefficient, and poor quality post-conflict health system [47]. In order to improve working conditions for HCWs, and incentivize return in this regard, national governments should coordinate with humanitarian NGOs, faith-based organizations, and initiatives led by expatriate physicians to effectively restore the health system [15, 46].

Based on the limited available literature, governments and international agencies interested in developing policies to facilitate the return and integration of conflict-displaced HCWs or propose policies to rehabilitate and reconstruct post-conflict health systems must first work towards guaranteeing a secure working environment for returning HCWs. Second, governments should carry out comprehensive assessment to identify gaps and priorities before implementing a national health policy framework that includes an explicit attention to return of conflict-displaced HCWs. Ideally, this should be part of a reconciliation process that brings information, clarity and reassurances for HCWs interested in return [54]. Third, governments must seek the views of and engage HCWs displaced by conflict regarding the opportunities and pathways for return and reintegration.

Given the limited number of post-conflict settings identified in this review, further research should be conducted in various post-conflict contexts in order to understand and address the particular barriers or enablers faced by conflict displaced HCWs in returning to their home country, including licensing and re-certification, social and psychological reintegration, employability within the post-conflict health system, and financial incentives to encourage HCW return. Future research should also explore whether HCWs displaced to contexts where they cannot or do not continue their training or work are more likely to return given the lack of employment opportunities, deterioration of their skills, and lack of ability to sustain livelihoods in their new host country contexts.

### Strengths & Limitations

To our knowledge, this is the first systematic review mapping policies and initiatives concerning the return and reintegration of HCWs to post-conflict settings. This has become an increasingly important issue as numbers of forcibly displaced populations have reached unprecedented highs in recent decades and conflict settings have become increasingly dangerous for HCWs.

There are potential limitations to this review. Our academic literature search was limited to articles which directly addressed policies regarding HCWs displaced by conflict. Relevant articles or reports may have been excluded or missed, including records published in languages other than English or Arabic. As a result, we found only a limited number of studies. No quality assessment was conducted to assess the methodological quality of the articles and reports included in the review. We focused on policies or initiatives specific to the health system, but clearly many factors beyond the health system influence decisions to return, including security issues and social and welfare policies. Finally, our review did not identify any explicit policies specific to HCWs returning to post-conflict settings, which limits the generalisability of our findings.

## Conclusion

The findings of this review raise central questions to a range of stakeholders in health systems including human resources for health researchers and policymakers investing in human resources for health development in post-conflict settings. By highlighting the limited attention given to formalizing and researching policies regarding the return and reintegration of displaced HCWs, the few records identified in this review provide a foundation for future research and policy development within and beyond the health sector regarding displaced HCW populations and provide implications for post-conflict governments as well as multilateral agencies coordinating and responding to increasing HCW shortages around the globe. Policies regarding health system rebuilding in conflict-affected contexts should go beyond the immediate focus of restoring disrupted health services and consider longer-term goals, such the return and reintegration of displaced HCWs and their contribution to the rehabilitation and reconstruction of post-conflict health systems.

## Data Availability

Not applicable.

## Appendix

*Search Syntax*

Syntax (Pubmed)

**Table:**

Search	Query	Items found 24 Jan 2020
#5	(#3 NOT #4)	4750
#4	“World War I”[Mesh] OR “World War II”[Mesh] OR world war*[tiab]	11,561
#3	(#1 AND #2)	5150
#2	post war [tiab] OR postwar [tiab] OR post conflict*[tiab] OR postconflict*[tiab] OR conflict affected [tiab] OR post genocid*[tiab] OR political transit*[tiab] OR fragile health system*[tiab] OR fragile state*[tiab] OR state building [tiab] OR ((conflict*[tiab] OR humanitarian*[tiab] OR war [tiab] OR crisis [tiab]) AND (recovery [tiab] OR reconstructing [tiab] OR reconstruction*[tiab] OR rebuild*[tiab] OR re build*[tiab] OR reform [tiab] OR reforms [tiab] OR reforming [tiab] OR reformed [tiab] OR reconcile [tiab] OR reconciles [tiab] OR reconciled [tiab] OR reconciling [tiab] OR reconciliation [tiab] OR rehabilitate [tiab] OR rehabilitates [tiab] OR rehabilitated [tiab] OR rehabilitating [tiab] OR rehabilitation [tiab] OR rehabilitations [tiab] OR re establish*[tiab] OR reintegrat*[tiab] OR re integrat*[tiab] OR capacity building [tiab] OR resource allocation [tiab]))	17,073
#1	“Health Personnel”[Mesh] OR “Health Workforce”[Mesh] OR ((“Health Services”[Mesh] OR health [tiab] OR healthcare [tiab] OR health care [tiab] OR medical [tiab] OR medically [tiab] OR clinical [tiab] OR clinically [tiab]) AND (personnel [tiab] OR worker*[tiab] OR provider*[tiab] OR workforce*[tiab] OR work force*[tiab] OR manpower [tiab] OR technician [tiab] OR technicians [tiab] OR consultant [tiab] OR consultants [tiab] OR staff [tiab] OR practitioner [tiab] OR practitioners [tiab] OR labor [tiab] OR labour [tiab] OR professional [tiab] OR professionals [tiab] OR “Education, Professional”[Mesh] OR student [tiab] OR students [tiab] OR trainee [tiab] OR trainees [tiab] OR intern [tiab] OR interns [tiab])) OR human resource*[tiab] OR local staff [tiab] OR clinic staff [tiab] OR clinic team*[tiab] OR doctor [tiab] OR doctors [tiab] OR clinician [tiab] OR clinicians [tiab] OR physician [tiab] OR physicians [tiab] OR dentist [tiab] OR dentists [tiab] OR pharmacist [tiab] OR pharmacists [tiab] OR nurse [tiab] OR nurses [tiab] OR nursing [tiab] OR nursings [tiab] OR midwife [tiab] OR midwives [tiab] OR midwifery [tiab] OR traditional birth attendant*[tiab] OR medical specialist [tiab] OR medical specialists [tiab] OR surgeon [tiab] OR surgeons [tiab] OR internist [tiab] OR internists [tiab] OR gynaecologist [tiab] OR gynaecologists [tiab] OR gynecologist [tiab] OR gynecologists [tiab] OR obstetrician [tiab] OR obstetricians [tiab] OR paediatrician [tiab] OR paediatricians [tiab] OR pediatrician [tiab] OR pediatricians [tiab] OR resident [tiab] OR residents [tiab] OR residency [tiab] OR psychiatrist [tiab] OR psychiatrists [tiab] OR psychologist [tiab] OR psychologists [tiab]	2,412,145

Syntax (Embase)

**Table:**

Search	Query	Items found 24 Jan 2020
#5	(#1 AND #2) NOT #4	5976
#4	‘world war*’:ti,ab	11,050
#3	(#1 AND #2)	6406
#2	‘post war’:ti,ab OR postwar:ti,ab OR ‘post conflict*’:ti,ab OR postconflict*:ti,ab OR ‘conflict affected’:ti,ab OR ‘post genocid*’:ti,ab OR ‘political transit*’:ti,ab OR ‘fragile health system*’:ti,ab OR ‘fragile state*’:ti,ab OR ‘state building’:ti,ab OR ((conflict*:ti,ab OR humanitarian*:ti,ab OR war:ti,ab OR crisis:ti,ab) AND (recovery:ti,ab OR reconstructing:ti,ab OR reconstruction*:ti,ab OR rebuild*:ti,ab OR ‘re build*’:ti,ab OR reform:ti,ab OR reforms:ti,ab OR reforming:ti,ab OR reformed:ti,ab OR reconcile:ti,ab OR reconciles:ti,ab OR reconciled:ti,ab OR reconciling:ti,ab OR reconciliation:ti,ab OR rehabilitate:ti,ab OR rehabilitates:ti,ab OR rehabilitated:ti,ab OR rehabilitating:ti,ab OR rehabilitation:ti,ab OR rehabilitations:ti,ab OR ‘re establish*’:ti,ab OR reintegrat*:ti,ab OR ‘re integrat*’:ti,ab OR ‘capacity building’:ti,ab OR ‘resource allocation’:ti,ab))	20,084
#1	‘health care personnel’/exp. OR ‘human resources’/exp. OR ((health:ti,ab OR healthcare:ti,ab OR ‘health care’:ti,ab OR medical:ti,ab OR medically:ti,ab OR clinical:ti,ab OR clinically:ti,ab) AND (personnel:ti,ab OR worker*:ti,ab OR provider*:ti,ab OR workforce*:ti,ab OR ‘work force*’:ti,ab OR manpower:ti,ab OR technician:ti,ab OR technicians:ti,ab OR consultant:ti,ab OR consultants:ti,ab OR staff:ti,ab OR practitioner:ti,ab OR practitioners:ti,ab OR labor:ti,ab OR labour:ti,ab OR professional:ti,ab OR professionals:ti,ab OR student:ti,ab OR students:ti,ab OR trainee:ti,ab OR trainees:ti,ab OR intern:ti,ab OR interns:ti,ab)) OR ‘human resource*’:ti,ab OR ‘local staff’:ti,ab OR ‘clinic staff’:ti,ab OR ‘clinic team*’:ti,ab OR doctor:ti,ab OR doctors:ti,ab OR clinician:ti,ab OR clinicians:ti,ab OR physician:ti,ab OR physicians:ti,ab OR dentist:ti,ab OR dentists:ti,ab OR pharmacist:ti,ab OR pharmacists:ti,ab OR nurse:ti,ab OR nurses:ti,ab OR nursing:ti,ab OR nursings:ti,ab OR midwife:ti,ab OR midwives:ti,ab OR midwifery:ti,ab OR ‘traditional birth attendant*’:ti,ab OR ‘medical specialist’:ti,ab OR ‘medical specialists’:ti,ab OR surgeon:ti,ab OR surgeons:ti,ab OR internist:ti,ab OR internists:ti,ab OR gynaecologist:ti,ab OR gynaecologists:ti,ab OR gynecologist:ti,ab OR gynecologists:ti,ab OR obstetrician:ti,ab OR obstetricians:ti,ab OR paediatrician:ti,ab OR paediatricians:ti,ab OR pediatrician:ti,ab OR pediatricians:ti,ab OR resident:ti,ab OR residents:ti,ab OR residency:ti,ab OR psychiatrist:ti,ab OR psychiatrists:ti,ab OR psychologist:ti,ab OR psychologists:ti,ab	3,462,907

Syntax (Web of Science)

**Table:**

Search	Query	Items found 24 Jan 2020
#5	(#3 NOT #4)	5939
#4	TOPIC: (“world war*”) Indexes = SCI-EXPANDED, SSCI, A&HCI, ESCI Timespan = All years	54,818
#3	(#1 AND #2)	6365
#2	**TOPIC:** (“post war” OR postwar OR “post conflict*” OR postconflict* OR “conflict affected” OR “post genocide*” OR “political transit*” OR “fragile health system*” OR “fragile state*” OR “state building” OR ((conflict* OR humanitarian* OR war OR crisis) AND (recovery OR reconstructing OR reconstruction* OR rebuild* OR “re build*” OR reform OR reforms OR reforming OR reformed OR reconcile OR reconciles OR reconciled OR reconciling OR reconciliation OR rehabilitate OR rehabilitates OR rehabilitated OR rehabilitating OR rehabilitation OR rehabilitations OR “re establish*” OR reintegrat* OR “re integrat*” OR “capacity building” OR “resource allocation”))) **Timespan:** All years. **Indexes:** SCI-EXPANDED, SSCI, A&HCI, ESCI.	79,576
#1	**TOPIC:** (((health OR healthcare OR “health care” OR medical OR medically OR clinical OR clinically) AND (personnel OR worker* OR provider* OR workforce* OR “work force*” OR manpower OR technician OR technicians OR consultant OR consultants OR staff OR practitioner OR practitioners OR labor OR labour OR professional OR professionals OR student OR students OR trainee OR trainees OR intern OR interns)) OR “human resource*” OR “local staff” OR “clinic staff” OR “clinic team*” OR doctor OR doctors OR clinician OR clinicians OR physician OR physicians OR dentist OR dentists OR pharmacist OR pharmacists OR nurse OR nurses OR nursing OR nursings OR midwife OR midwives OR midwifery OR “traditional birth attendant*” OR “medical specialist” OR “medical specialists” OR surgeon OR surgeons OR internist OR internists OR gynaecologist OR gynaecologists OR gynecologist OR gynecologists OR obstetrician OR obstetricians OR paediatrician OR paediatricians OR pediatrician OR pediatricians OR resident OR residents OR residency OR psychiatrist OR psychiatrists OR psychologist OR psychologists) **Timespan:** All years. **Indexes:** SCI-EXPANDED, SSCI, A&HCI, ESCI.	1,896,732

## Abbreviations

CHWs: Community Healthcare Workers
HCWs: Healthcare Workers
IOM: International Organization for Migration
MoPH: Ministry of Public Health
NGOs: Non-Governmental Organizations
ODI: Overseas Development Institute
PRISMA: Preferred Reporting Items for Systematic Reviews and Meta-Analyses
SPRP: Sudanese Physician Reintegration Program
UN: United Nations
UNHCR: United Nations High Commissioner for Refugees
U.S.: United States
WHO: World Health Organization
